# Dental Students in Germany throughout the COVID-19 Pandemic: A Psychological Assessment and Cross-Sectional Survey

**DOI:** 10.3390/biology10070611

**Published:** 2021-07-01

**Authors:** Mohamed Mekhemar, Sameh Attia, Christof Dörfer, Jonas Conrad

**Affiliations:** 1 Clinic for Conservative Dentistry and Periodontology, School of Dental Medicine, Kiel University, Arnold-Heller-Str. 3, Haus B, 24105 Kiel, Germany; doerfer@konspar.uni-kiel.de; 2 Department of Oral and Maxillofacial Surgery, Justus-Liebig University Giessen, Klinik Str. 33, 35392 Giessen, Germany; sameh.attia@dentist.med.uni-giessen.de

**Keywords:** COVID-19, IES-R, DASS-21, anxiety, stress, depression, dental students, psychological impact, mental health

## Abstract

**Simple Summary:**

The Coronavirus Disease (COVID-19) outbreak has presented severe public health risks throughout the world since 2019. Among various health impacts of the pandemic globally, it has led to unprecedented risks to the mental wellbeing of healthcare professionals and students. This study aimed to examine the impact of the pandemic and several demographic and social factors on dental students’ mental health in German universities nationwide from July 2020 to January 2021. The survey assembled data through an online platform on demographics, the Depression Anxiety Stress Scales (DASS-21), and the Impact of Events Scale-Revised (IES-R) instrument from 211 participants and examined correlations between all variables and mental consequences of depression, anxiety, stress, intrusion, avoidance, and hyperarousal in the study population. Dental students showed an overall mild impact of the outbreak on the assessed psychological aspects. Female students and students having cardiovascular conditions or smoking habitually, as well as participants perceiving COVID-19 as a financial danger were significantly affected more by the disease than their counterparts. Highlighting these results can help to protect dental students’ mental health in German universities during the crisis and support the authorities to adjust the required actions to counteract detrimental effects of the outbreak

**Abstract:**

Multiple investigations have reported high psychological distress among students since the coronavirus (COVID-19) outbreak started. This survey examined the associations between psychological features, and several demographic and social factors among dental students in German universities. Dental students registered in German universities nationwide were asked to join this survey via a self-directed online questionnaire, from July 2020 to January 2021. This study assembled data on demographic statistics, the depression anxiety stress scales (DASS-21), and the impact of events scale—revised (IES-R) instrument. The relationships between demographic-related variables and mental consequences of depression, anxiety, stress, intrusion, avoidance, and hyperarousal were inspected. Two hundred and eleven students contributed to the questionnaire and conveyed overall normal or mild outcomes of depression, anxiety, stress, intrusion, avoidance, and hyperarousal. In addition, female gender, cardiovascular diseases, smoking habits, and seeing the COVID-19 outbreak as a financial risk were stated as significant related factors (*p* < 0.05), with increased IES-R and DASS-21 scores. These results highlight the features that should be considered to better protect dental students’ mental health in German universities during the crisis.

## 1. Introduction

In December 2019, the city of Wuhan in China became the epicenter of a pneumonia epidemic of uncertain origin, later recognized as SARS-CoV-2 and named, by the World Health Organization, as the 2019 novel coronavirus (COVID-19). Because of its high virulence and mortality, this strain was declared a worldwide pandemic on March 11 2020. Its increased infection rate, along with a rising number of associated severe health deterioration or deaths, sparked intense public unrest and panic. Early research on the immediate mental outcomes of the current outbreak on the general population identified mild to extreme psychological consequences of the disease [1,2].

In addition to its psychological impact on the general population, the COVID-19 pandemic has challenged and often surpassed the capacity of health professionals and facilities worldwide. Healthcare workers had to continue providing care for patients in spite of physical and mental exhaustion, high risk of infection, their fear to transmit the disease to their loved ones, and the loss of many patients. Additionally, long working shifts combined with heavy restrictions, together with personal isolation, have affected the mental status of various health professionals [3]. Similar to other medical branches, the COVID-19 outbreak hindered the efforts of dental healthcare professionals and students [4,5]. Multiple dental procedures have been suspended due to the increased risk of viral cross-infection during treatment, limiting dental visits only to emergency cases [6].

Dental manufacturers and several dental practices have suspended some employees, as an outcome of the financial pressure during the pandemic [7]. In fact, dentistry was classified among other medical branches as a high-risk profession for the likely occupational virus transmission, due to the close proximity of dental healthcare personnel to the patient throughout the treatment, and the procedural generation of infective aerosols [8]. Dentistry students, in specific, are considered at a higher risk than all other dental healthcare workers for COVID-19 infection, owing to their inexperience, practical insufficiency, and deficiency of knowledge [9]. Besides their worries about cross-infection among their patients and families, the social implications of lockdowns and the discontinuing or rapid changes in scientific or educational programs might present effective sources of mental distress among the students [4,6,9].

In Germany, the current pandemic has resulted in over one million cases of COVID-19 infections, and multiple deaths. Though COVID-19-related deaths are relatively uncommon in Germany’s younger age groups, they account for a significant portion of COVID-19-related hospitalizations [10,11,12]. Furthermore, during the first wave of outbreaks in 2020, researchers discovered that people between 20 and 24 years of age play a key role in spreading the SARS-CoV-2 infection in Germany [13]. This implies that university students, the majority of whom are in this age group, may play a crucial role in SARS-CoV-2 transmission [12]. Furthermore, during the second wave of infection in the summer and autumn of 2020, individuals of the student group were found to be more prevalent among those infected, confirming the importance of observing and understanding this group and its circumstances during the pandemic [12]. With about 80.5 million people, almost 70,000 dentists, and 32 dental schools, Germany, the most populous nation of the European Union, is among the largest European states in terms of the number of oral health professionals and students [14,15]. In recent years, the mental wellbeing of dental and medical students, particularly during difficult situations, has been a growing concern worldwide, with an estimation of potential mental disorders in one in every five students [16]. Although earlier investigations reported the psychological distress affecting German dentists throughout the outbreak [6], the psychological consequences on dental students, as one of the main dental service providers in university clinics and future dental professionals [15], accompanying the crisis and its key aspects, still need to be disclosed. Thus, this investigation intended to explore this subject via the impact of event scale—Revised (IES-R) and depression anxiety stress scale (DASS-21) examinations on a national scale among Germany’s dental students.

## 2. Materials and Methods

### 2.1. Study Population and Procedures

A national cross-sectional study of dental students in Germany was conducted to assess the psychological outcome of COVID-19 and its associated causes. An online survey was developed using an internet-based survey platform to reduce face-to-face communication and encourage contribution by all students (Unipark, QuestBack GmbH, Cologne, Germany). Following the University of Kiel Ethics Board (D452/18) approval, a link for the online survey was posted on numerous social network pages associated with dentistry and invitations were sent to registered dental students from various universities in Germany, as performed in previous studies [15]. The survey’s introductory text quickly clarified the study and the voluntary and anonymous involvement of the students. There were no financial benefits assured to participating students, and no exclusion requirements were established (e.g., gender, age, or nationality). At the start of the survey, students consented, indicating their willingness to participate. Data collection was completed from July 2020 to January 2021.

### 2.2. Survey Instruments

The primary segment of the survey gathered sociodemographic data from respondents such as their age, federal state, parenthood, marital status, systemic comorbidities, and smoking status. Students also stated if they believed the outbreak represented a private financial risk.

Following up on that point, in the second portion of the questionnaire, participants received the depression anxiety stress scale (DASS-21) and impact of event scale—revised (IES-R) tool. Both survey instruments were applied with their scoring systems and cut-off scores to evaluate depression, anxiety, stress, as well as intrusion, avoidance, and hyperarousal as applied for German dentists previously [6]. The DASS-21 subscales were scored as follows: normal (0–4 DASS-21 points), mild (5–6 DASS-21 points), moderate (7–10 DASS-21 points), severe (11–13 DASS-21 points), and extremely severe (14+ DASS-21 points) for depression; normal (0–3 DASS-21 points), mild (4–5 DASS-21 points), moderate (6–7 DASS-21 points), severe (8–9 DASS-21 points), and extremely severe (10+ DASS-21 points) for anxiety; and normal (0–7 DASS-21 points), mild (8–9 DASS-21 points), moderate (10–12 DASS-21 points), severe (13–16 DASS-21 points), and extremely severe (17+ DASS-21 points) for stress. The IES-R subscores were categorized as normal (0–23 IES-R points), mild (24–32 IES-R points), moderate (33–36 IES-R points), and severe psychological impact of events (>37 IES-R points). Both survey instruments were previously validated in their German version and displayed a significant reliability to measure the psychological impact of the COVID-19 pandemic on general populations and healthcare personnel [6].

### 2.3. Sample Size Calculation

To decide how many responses were required for a significant size of the sample population, conditions were defined analogously to earlier investigations on dental students in Germany [15] for the sample size calculation.

The following conditions were defined:

1. Number of sent emails to registered students (*N* = 207);
2. A confidence level of 95%;
3. A margin of error of 5%.

Based on this calculation, it was decided that at least 135 dental students were required for a sufficient study group across Germany.

### 2.4. Statistical Analysis

Outcomes were digitally recorded by the online-based survey instrument and analyzed afterwards by SPSS software (SPSS Statistic 27, IBM, Armonk, NY, USA). Descriptive analysis was completed and the normality of all data variables was tested by the Kolmogorov and Shapiro–Wilk test. Data of all scales and sub-scales showed no normal distribution. No transformations of variables were attempted for normalization based on the statistical analysis performed previously on German dentists [6]. Skewed distributions of variables have also been reported to be commonly used and accepted in psychological and social research and do not require normalization procedures necessarily [17,18]. To examine the relations between DASS-21/IES-R ratings and sociodemographic factors, univariate analyses (Kruskal–Wallis and Mann–Whitney U test) were applied. For significant statistical test outcomes post hoc, the Dunn–Bonferroni statistical test was further applied in single comparisons. Then multiple linear regression tests were executed on DASS-21 total and subscores and the IES-R subscales to distinguish the involvement of the earlier recognized, relevant factors. Statistical significance was set at *p* < 0.05.

## 3. Results

### 3.1. Participation and Sociodemographic Data

An overall number of 211 dental students contributed to the study, displaying a sample size that was sufficient for the required statistical power. The students encompassed female (73.5%), male (26%), and third-gender dental students (0.5%) from all dental schools in Germany. All of the respondents were younger than 50 years old. Most of the participating students were single (81.5%) and did not have any children (96.7%), while the rest were either in a marriage or marriage-like relationship (18.5%), and were responsible for children (3.3%). Around one-third of the participants (32.7%) considered the COVID-19 outbreak to be a danger to their financial stability. Additionally, the study group exhibited a 5.7% rate of smoking and displayed multiple medical comorbidities, with 3.3% cardiovascular diseases, 2.4% chronic pulmonary conditions, and 0.9% immunodeficiencies (Table 1).

### 3.2. DASS-21 and IES-R Scales and Associated Factors

The inspection of psychological indicators in the study sample, by the DASS-21 and IES-R scales, are displayed in Table 2 and Table 3, and in relation to the associated aspects in Table 4 and Table 5, respectively.

The applied grading system exhibited that the total survey population of dental students displayed DASS-21 and IES-R scores of normal psychological behaviors, with potential slight distress due to the outbreak (Table 2 and Table 3).

The DASS-21 and IES-R scales-related factors presented statistically higher DASS-21, and IES-R total and subscale outcomes, labelling mild to normal mental consequences amongst dental students of the female or third gender, having cardiovascular diseases, with a smoking habit, as well as students seeing the outbreak as a financial danger (Table 4 and Table 5).

Multiple regression analyses of DASS-21 total and subscores within the study model showed a significant impact of financial factors, systemic cardiovascular diseases, the participants ‘gender, and habitual smoking on the psychological stress, depression, and anxiety, as well as intrusion, avoidance, and hyperarousal of German dental students (Table 6 and Table 7).

## 4. Discussion

The first verified COVID-19 infection was reported in Bavaria, Germany, in the early months of 2020 [19]. Fast adjustment processes were implemented by the German healthcare facilities, and rapid efforts were made to respond to the outbreak. This crisis had an inescapable impact on healthcare societies and educational institutions across the country [20,21]. The dental society is among the most affected by the pandemic, both internationally and within Germany, due to a variety of reasons impacting its members’ psychological well-being and financial security during the crisis and its associated lockdowns [6]. So far, this survey is the first to assess the mental effect of the COVID-19 outbreak on dental students in Germany, countrywide, by the DASS-21 and IES-R survey tools as major screening instruments used globally for healthcare workers and student psychological evaluations.

In this study, 211 dental students participated nationwide, through the web-based survey link. As they finished the online survey, the study population displayed a significant sample size, representing Germany’s dental students in the study. In comparison to prior surveys on dental students in Germany (Table 1), the respondents’ sociodemographic data revealed a similar gender distribution, with larger percentages of female students [15]. Unsurprisingly, all of the survey contributors presented an age less than 50 years (Table 1), corresponding to the described typical age (20–50 years) among medical university students in Germany [22]. Consequently, the total study population did not express previously described age-related COVID-19 risk factors [6]. Furthermore, the survey participants reported a smoking rate of nearly 6% (Table 1), similar to the conveyed outcomes of dental professionals in Germany (5–8%), in addition to other oral health societies globally [15]. Cardiovascular diseases displayed the major systemic diseases among a small group of the participating students (Table 1), which is consistent with the results stated by former reports on Germany’s population [23].

In the present survey, dental students showed an overall normal or mild psychological impact of the pandemic on anxiety, stress, depression, intrusion, hyperarousal and avoidance, as assessed by the explained survey instruments. This displays a discrepancy to the outcomes of similar studies on healthcare professionals and dental or medical students in other non-European countries, which showed higher levels of psychological distress [4,6,24,25,26,27,28], and could represent the psychological significance of Germany’s claimed success in containing COVID-19 infection rates through introducing new safety regulations in health and educational institutions, stabilizing its population’s financial situation throughout the crisis, and communicating the reasons for its emergency policies [6,29,30,31]. Furthermore, several variables distressing students in German universities throughout the outbreak appear to actively participate in the development of stress-, anxiety-, depression- and PTSD-associated symptoms. Similar to previous reports on German dentists [6], the female students in the current survey presented significantly higher anxiety and stress, as well as IES-R subscales (Table 4 and Table 5) than the male participants. This situation is explained by earlier findings that females are statistically more likely than men to acquire mental distress, depressive symptoms, and PTSD signs as adults [29,30]. This variance between the two genders was described as a result of different thought control approaches during difficult life situations, and discrepant metacognitive beliefs among males and females, promoting an intensified mental effect among the latter ones [30]. While only 0.5% of the study sample characterized third-gender members of the dental students (Table 1), the single respondent showed intense psychological distress compared to other genders (Table 4 and Table 5). This outcome also approves the results seen among third-gender German dentists [6] and reported social tensions, leading to mental health concerns among this part of the society [32]. The current investigation further reported that aspects among the participants, such as being single and having no child responsibilities, were linked to healthier mental outcomes than being in a marriage relationship or raising children (Table 4 and Table 5). This consequence was observed similarly among the German population during the COVID-19 crisis, as individuals with no relationship status had a significantly more stable mental health than married couples or individuals living with a partner, specifically in cases with poor relationship quality [33]. Amongst multiple factors, the economic strain created by the COVID-19 lockdowns might be closely related to a decline in the marital and partnership quality of the affected individuals [34]. Moreover, previous studies on parents’ mental health have described how early parenthood often promotes psychological distress and depression [35], which can be associated with the students’ in early paternity and motherhood.

Systemic disorders have been previously linked to the higher morbidity and mortality rates of the affected patients during the pandemic [36,37]. As a result, those who have been afflicted conveyed more psychological distress [1]. In the current survey, dental students in Germany have exhibited a similar outcome during the COVID-19 pandemic, particularly among vascular diseases (Table 4 and Table 5). This also confirms earlier results reported on German dentists, which have displayed cardiovascular disease as one of the significant factors of mental decline during the pandemic [6]. Indeed, underlying cardiovascular comorbidities are associated with high mortality rates of COVID-19 patients. Moreover, a COVID-19 infection can even induce or deteriorate cardiovascular conditions, such as myocardial infarctions, arrhythmias and venous thromboembolism [38,39]. Hypertension was furthermore described to have the highest prevalence as a comorbidity in COVID-19 patients [40], creating an obvious stress factor for cardiovascular patients [41]. Another comorbidity increasing the risk of COVID-19-associated complications is pulmonary disease [42]. As reported previously, patients having pulmonary conditions presented high incidences of depression, COVID-19-related stress, post-traumatic symptoms, as well as insomnia during the lockdown [43]. Furthermore, as smoking is considered a main risk factor for malignant and nonmalignant conditions of the respiratory tract [44,45], significantly higher mental health distress was noted among the smoking participants of the current survey (Table 4, Table 5, Table 6 and Table 7). This conforms to multiple studies describing the higher mental distress among smokers during the pandemic. These reports even observed higher rates of smoking among the affected individuals, leading to a vicious cycle of mental health deterioration and habitual smoking [46,47,48,49].

Having to face the unexpected consequences during the pandemic, students, predominantly in the medical field, were among the most affected groups globally, showing increased levels of stress, anxiety, and depression [50,51,52]. With elevated financial insecurity after losing employment, besides the anxiety about the digitalized home-schooling or delayed graduation, students had numerous difficulties getting through the lockdown and the imposed regulations [51,53]. This outcome was also observable among dental students in Germany, as the respondents that considered COVID-19 as threatening their financial stability had significantly higher scores in the current survey (Table 4, Table 5, Table 6 and Table 7). However, this financial pressure might show variances between different German states and regions. Recent studies described the socioeconomic disparities within the German population, based on the cities and living areas [54]. On the other hand, due to the predominantly government- and tax-funded dental and medical education and its services in German universities, and the very similar life standards among the majority of German university students, this socioeconomic discrepancy might be less significant on the student level [15,55]. As reported previously, severe restrictions were additionally enforced, particularly on the practical training component of undergraduate dental medical training, creating new challenges for the students and universities in adapting to the new digital platforms and education techniques [20,21]. Moreover, many medical and dental students from German universities displayed higher stress aspects during the pandemic, in terms of being worried about the continuation or quality of their education [56,57]. Interestingly, some investigations have observed an enhancement of students’ learning performance during the COVID-19 lockdowns [58]. This was further explained, as students may be driven by their inherent responsibility in confusing situations to contribute as much as they can to solve the difficulties that education is experiencing during the pandemic. This would lead different students to find different motivations to guarantee good and safe progress in this academic year, despite the outbreak [58].

Multiple linear regression tests were performed to determine independent outcomes of the significant analyzed factors of the pandemic as a financial risk, participant gender, smoking status, and systemic comorbidities on the survey instrument scores. Female gender, being a smoker or cardiovascular patient, and seeing the pandemic as an economic burden were independently linked with worse psychological outcomes (Table 4, Table 5, Table 6 and Table 7), emphasizing these aspects as the most effective on dental students’ psychological stability in Germany throughout the COVID-19 pandemic.

## 5. Limitations

To date, this is the first study to examine the mental effect of the COVID-19 pandemic on dental students countrywide in Germany, by using the DASS-21 and IES-R survey instruments. Yet, some limiting aspects to the survey have to be described. Firstly, the study is limited, as it is cross-sectional and has no longitudinal follow-up parts. Our current outcomes of dental students cannot be ascribed entirely to the observed aspects and socio-environmental information. Unrecorded factors (as the exact financial status) could be an essential factor in changing some survey interpretations.

Furthermore, the survey participation was performed during several months. Due to the high sensitivity of the COVID-19 crisis and the rapid fluctuations in the infection rates and applied protocols, such changes could impact the results described by the study participants as well. Additionally, having a voluntary study might produce an effect of selection bias amongst participants. Lastly, to reduce face-to-face contact and maximize the participation, an online self-report questionnaire was applied, to evaluate the psychological symptoms without a diagnostic evaluation by mental health specialists. An addition of a clinical assessment by professionals of psychology would undoubtedly add an important feature to the survey. Notwithstanding the above limitations, the displayed conclusions of this study present essential and unique data on the psychological effect of the ongoing pandemic on dental students in German universities.

## 6. Conclusions

Dental students’ mental health is imperative for their educational and behavioral development during the COVID-19 crisis, and their subsequent careers. Our conclusions exhibited that female gender, being chronically ill with cardiovascular conditions, habitually smoking, and seeing COVID-19 as a financial risk are statistically significant aspects leading to a psychological decline among dental students across Germany during the pandemic, and these factors increased IES-R and DASS-21 outcomes. Examining such factors closely could support Germany’s educational and health institutions to adjust the implementation of required actions to minimize the psychological harm of the outbreak on Germany’s future dentists.

## Figures and Tables

**Table 1:** Characteristics of participants (*n* = 211).

Sociodemographic Factors		N	%
**Gender**			
	Female	155	73.5
	Male	55	26.0
	Third Gender	1	0.5
**Age**			
	18–49	211	100.0
	50–59	0	0
	≥60	0	0
**Marital status**			
	Single	172	81.5
	Married or in a marriage-like partnership	39	18.5
	Divorced, separated, or widowed	0	0
**Having children**			
	Yes	7	3.3
	No	204	96.7
**COVID-19 being a personal financial threat**			
	Yes	69	32.7
	No	142	67.3
**Smoker**			
	Yes	12	5.7
	No	199	94.3
**Medical comorbidity ^1^**			
	Diseases of the cardiovascular system (e.g., coronary heart disease and high blood pressure)	7	3.3
	Chronic lung diseases (e.g., COPD)	5	2.4
	Chronic liver diseases	0	0
	Diabetes mellitus	0	0
	Cancer	0	0
	Immunodeficiency	2	0.9

^1^ No or multiple choice was possible.

**Table 2:** DASS-21 and IES-R scores of the study sample.

Psychological Evaluation		Mean ± SD	Interquartile Range
**DASS-21 (*n* = 211) ^1^**			
	Total	11.74 ± 10.03	13
	Depression	4.01 ± 3.93	5
	Anxiety	2.52 ± 3.20	4
	Stress	5.21 ± 4.09	6
**IES-R (*n* = 204) ^1^**			
	Intrusion	5.27 ± 5.54	7
	Avoidance	7.74 ± 7.40	11
	Hyperarousal	6.21 ± 6.07	9

^1^ *n* varies because of missing data.

**Table 3:** The number of dental students and total population percentage for each DASS-21 and IES-R subscale category.

	Subscale	Category	N	%
**DASS-21** (*n* = 211) ^1^				
	Depression	normal	134	63.5
		mild	37	17.5
		moderate	24	11.4
		severe	9	4.3
		extremely severe	7	3.3
	Anxiety	normal	155	73.5
		mild	26	12.3
		moderate	11	5.2
		severe	9	4.3
		extremely severe	10	4.7
	Stress	normal	157	74.4
		mild	21	10.0
		moderate	20	9.5
		severe	10	4.7
		extremely severe	3	1.4
**IES-R** (*n* = 204) ^1^				
	Intrusion	normal	204	100.0
		mild	0	0
		moderate	0	0
		severe	0	0
	Avoidance	normal	196	96.1
		mild	6	2.9
		moderate	2	1.0
		severe	0	0
	Hyperarousal	normal	202	95.7
		mild	2	0.9
		moderate	0	0
		severe	0	0

^1^ *n* varies because of missing data.

**Table 4:** Differences between participants’ characteristics regarding DASS-21 total and subscale scores.

	DASS-21 Total			DASS-21 Depression			DASS-21 Anxiety			DASS-21 Stress		
	Mean ± SD	Test Statistic	*p*-Value	Mean ± SD	Test Statistic	*p*-Value	Mean ± SD	Test Statistic	*p*-Value	Mean ± SD	Test Statistic	*p*-Value
**Gender**												
Female	12.57 ± 9.94	H = 10.81	< 0.01	4.19 ± 3.76	H = 8.08	0.02	2.75 ± 3.26	H = 10.64	0.01	5.63 ± 4.08	H = 11.22	<0.01
Male	8.82 ± 8.91			3.31 ± 4.05			1.67 ± 2.67			3.84 ± 3.62		
Third Gender	44.00 ± 0.00			16.00 ± 0.00			12.00 ± 0.00			16.00 ± 0.00		
Post hoc: Dunn–Bonferroni Test												
Male–Female		27.08	0.01		21.94	0.02		26.16	0.01		27.65	<0.01
Male–Third G		−122.47	0.05		−117.17	0.06		−119.78	0.05		−122.89	0.05
Female–Third G		−95.39	0.12		−95.23	0.12		−93.63	0.12		−95.25	0.12
**Age**												
18–49	11.74 ± 10.03	-	-	4.01 ± 3.93	-	-	2.52 ± 3.20	-	-	5.21 ± 4.09	-	-
50–59	-			-			-			-		
≥60	-			-			-			-		
**Marital status**												
Single	11.50 ± 9.96	H = 0.50	0.48	3.98 ± 4.03	H = 0.58	0.45	2.39 ± 2.97	H = 0.24	0.62	5.13 ± 4.12	H= 0.56	0.46
Married or in a marriage-like partnership	12.82 ± 10.41			4.15 ± 3.49			3.08 ±4.06			5.59 ± 4.00		
Divorced, separated, or widowed	-			-			-			-		
**Having children**												
Yes	16.57 ± 14.54	U = 586.50	0.42	6.29 ± 6.05	U = 564.50	0.34	4.43 ± 4.72	U = 544.00	0.27	5.86 ± 4.60	U = 653.00	0.70
No	11.58 ± 9.85			3.94 ± 3.83			2.45 ± 3.14			5.19 ± 4.08		
**COVID-19 being a personal financial threat**												
Yes	13.71 ± 10.76	U = 4073.50	0.05	4.86 ± 4.46	U = 4065.50	0.04	2.99 ± 3.39	U = 4259.50	0.12	5.87 ± 4.05	U = 4122.50	0.06
No	10.79 ± 59.55			3.61 ±3.59			2.29 ±3.09			4.89 ± 4.09		
**Smoker**												
Yes	18.83 ± 13.74	U = 775.50	0.04	6.92 ± 5.57	U = 762.50	0.03	4.25 ± 5.07	U = 894.50	0.14	7.67 ± 4.16	U = 761.50	0.03
No	11.32 ± 9.64			3.84 ± 3.75			2.41 ± 3.04			5.07 ± 4.05		
**Medical comorbidity (Multiple Choice)**												
No medical comorbidities	11.43 ± 9.62			3.92 ± 3.78			2.38 ± 3.00			5.13 ± 4.04		
Diseases of the cardiovascular system (e.g., coronary heart disease and high blood pressure)	24.71 ± 16.00	U = 1084.00	0.02	8.57 ± 6.43	U = 1038.00	0.04	7.57 ± 5.47	U = 1140.50	0.01	8.57 ± 5.53	U = 1000.50	0.07
Chronic lung diseases (e.g., COPD)	7.60 ± 5.73	U = 397.50	0.38	2.40 ± 1.95	U = 410.00	0.43	1.60 ± 1.67	U = 466.50	0.71	3.60 ± 2.97	U = 408.00	0.43
Chronic liver diseases	-	-	-	-	-	-	-	-	-	-	-	-
Diabetes mellitus	-	-	-	-	-	-	-	-	-	-	-	-
Cancer	-	-	-	-	-	-	-	-	-	-	-	-
Immunodeficiency	7.50 ± 0.71	U = 171.50	0.66	1.50 ± 0.71	U = 132.00	0.37	0.50± 0.71	U = 123.50	0.31	5.50 ± 0.71	U = 237.00	0.74

SD = standard deviation; H = test statistic of Kruskal–Wallis test; U = test statistic of Mann–Whitney U test; significant results are highlighted.

**Table 5:** Differences between participants’ characteristics regarding IES-R subscale scores.

	IES-R Intrusion			IES-R Avoidance			IES-R Hyperarousal		
	Mean ± SD	Test Statistic	*p*-Value	Mean ± SD	Test Statistic	*p*-Value	Mean ± SD	Test Statistic	*p*-Value
**Gender**									
Female	6.03 ± 5.90	H = 10.97	<0.01	8.55 ± 7.47	H = 9.54	<0.01	6.87 ± 6.26	H = 8.50	<0.01
Male	3.08 ± 3.55			5.37 ± 6.71			4.29 ± 5.07		
Third Gender	-			-			-		
**Age**									
18–49	5.27 ± 5.54			7.74 ± 7.40			6.21 ± 6.07		
50–59	-			-			-		
≥60	-			-			-		
**Marital status**									
Single	5.19 ± 5.48	H = 0.07	0.79	7.47 ± 7.11	H = 0.33	0.57	5.87 ± 5.99	H = 2.71	0.10
Married or in a marriage-like partnership	5.64 ± 5.86			8.85 ± 8.53			7.67 ± 6.27		
Divorced, separated, or widowed	-			-			-		
**Having children**									
Yes	6.29 ± 5.65	U = 590.00	0.51	10.14 ± 9.53	U = 582.50	0.48	8.43 ± 7.23	U = 562.00	0.40
No	5.24 ± 5.55			7.65 ± 7.33			6.13 ± 6.03		
**COVID-19 being a personal financial threat**									
Yes	6.21 ± 5.45	U = 3692.00	0.03	9.17 ± 8.11	U = 3910.50	0.10	8.20 ± 6.30	U = 3166.50	<0.01
No	4.83 ± 5.55			7.05 ± 6.96			5.26 ± 5.74		
**Smoker**									
Yes	6.67 ± 7.80	U = 1097. 50	0.78	10.00 ± 9.82	U = 1030.00	0.54	12.00 ± 8.33	U = 641.00	0.01
No	5.19 ± 5.39			7.59 ± 7.23			5.85 ± 5.74		
**Medical comorbidity (Multiple Choice)**									
No medical comorbidities	5.14 ± 5.45			7.68 ± 7.38			6.03 ± 5.92		
Diseases of the cardiovascular system (e.g., coronary heart disease and high blood pressure)	11.17 ± 7.91	U = 877.00	0.05	13.67 ± 9.00	U = 850.50	0.07	14.17 ± 8.31	U = 934.00	0.02
Chronic lung diseases (e.g., COPD)	4.00 ± 3.24	U = 481.50	0.90	3.20 ± 3.11	U = 324.50	0.18	4.40 ± 4.04	U = 435.50	0.63
Chronic liver diseases	-	-	-	-	-	-	-	-	-
Diabetes mellitus	-	-	-	-	-	-	-	-	-
Cancer	-	-	-	-	-	-	-	-	
Immunodeficiency	4.00 ± 1.41	U = 212.00	0.90	7.00 ± 1.41	U = 225.50	0.78	4.00 ± 0.00	U = 193.00	0.91

SD = standard deviation; H = test statistic of Kruskal–Wallis test; U = test statistic of Mann–Whitney U test; significant results are highlighted.

**Table 6:** Multiple regression analyses with relevant factors of DASS-21 total and subscores.

	B	SE	β	T	p	95% CI
**DASS-21 Total**						
Gender ^1^	−3.06	1.46	−0.14	−2.09	0.04	−5.93; −0.18
COVID-19 being personal financial threat ^2^	−2.30	1.41	−0.11	−1.64	0.10	−5.07; 0.47
Smoker ^2^	−6.85	2.86	−0.16	−2.34	0.02	−12.47; −1.22
Medical comorbidity: diseases of the cardiovascular system (e.g., coronary heart disease and high blood pressure) ^3^	13.52	3.70	0.24	3.65	<0.01	6.22; 20.81
**DASS-21 Depression**						
Gender ^1^	−0.63	0.58	−0.07	−1.09	0.28	−1.77; 0.51
COVID-19 being personal financial threat ^2^	−1.04	0.56	−0.12	−1.86	−0.06	−2.13; 0.06
Smoker ^2^	−2.74	1.13	−0.16	−2.43	0.02	−4.97; −0.51
Medical comorbidity: diseases of the cardiovascular system (e.g., coronary heart disease and high blood pressure) ^3^	4.56	1.46	0.21	3.11	<0.01	1.67; 7.44
**DASS-21 Anxiety**						
Gender ^1^	−0.90	0.47	−0.13	−1.93	0.06	−1.81; 0.02
Medical comorbidity: diseases of the cardiovascular system (e.g., coronary heart disease and high blood pressure) ^3^	5.51	1.18	0.31	4.66	<0.01	3.18; 7.84
**DASS-21 Stress**						
Gender ^1^	−0.63	0.48	−0.09	−1.30	0.20	−1.58; 0.33

B = unstandardized beta coefficient; SE = standard error; β = standardized beta coefficient; *p* = *p*-value; CI: confidence interval; significant results are highlighted; ^1^ 1 = female; 2 = male; 3 = third gender; ^2^ 1 = yes; 2 = no; ^3^ 0 = not quoted; 1 = quoted.

**Table 7:** Multiple regression analyses with relevant factors of IES-R scores.

IES-R Intrusion	B	SE	β	T	p	95% CI
Gender ^1^	−2.92	0.86	−0.23	−3.41	<0.01	−4.60; −1.23
COVID-19 being personal financial threat ^2^	−1.13	0.80	−0.10	−1.42	0.16	−2.70; 0.44
Medical comorbidity: diseases of the cardiovascular system (e.g., coronary heart disease and high blood pressure) ^3^	6.30	2.20	0.19	2.87	0.01	1.97; 10.62
**IES-R Avoidance**						
Gender ^1^	−3.18	1.17	−0.19	−2.72	0.01	−5.49; −0.87
**IES-R Hyperarousal**						
Gender ^1^	−2.72	0.90	−0.20	−3.03	<0.01	−4.50; -0.95
COVID-19 being personal financial threat ^2^	−2.42	0.84	−0.19	−2.89	<0.01	−4.07; −0.77
Smoker ^2^	−5.72	1.67	−0.22	−3.42	<0.01	−9.02; −2.42
Medical comorbidity: Diseases of the cardiovascular system (e.g., coronary heart disease and high blood pressure) ^3^	7.76	2.31	0.22	3.36	<0.01	3.21; 12.31

B = unstandardized beta coefficient; SE = standard error; β = standardized beta coefficient; p = *p*-value; CI: confidence interval; significant results are highlighted; ^1^ 1 = female; 2 = male; 3 = third gender; ^2^ 1 = yes; 2 = no; ^3^ 0 = not quoted; 1 = quoted.

## Data Availability

The data presented in this study are available on request from the corresponding author.
